# Histology and Immunohistochemistry of Radial Arteries Are Suggestive of an Interaction between Calcification and Early Atherosclerotic Lesions in Chronic Kidney Disease

**DOI:** 10.3390/medicina57111156

**Published:** 2021-10-24

**Authors:** Aikaterini Lysitska, Nikiforos Galanis, Ioannis Skandalos, Christina Nikolaidou, Sophia Briza, Asimina Fylaktou, George Lioulios, Zoi Mitsoglou, Dorothea Papadopoulou, Nikolaos Antoniadis, Aikaterini Papagianni, Maria Stangou

**Affiliations:** 1 Department of Nephrology, Papageorgiou General Hospital, 56429 Thessaloniki, Greece; lyskaterina@yahoo.gr (A.L.); dorapagia987@gmail.com (D.P.); 2 Department of Orhtopediscs, Papanikolaou General Hospital, Aristotle University of Thessaloniki, 57010 Thessaloniki, Greece; ngalanismed@gmail.com; 3 Department of Surgery, Agios Pavlos General Hospital, 55134 Thessaloniki, Greece; ioannisskandalos@yahoo.gr; 4 Department of Pathology, Hippokration General Hospital, 54642 Thessaloniki, Greece; christinapathologist@gmail.com; 5 Departure of Architecture, School of Engineering, University of Thessaly, 38334 Thessaly, Greece; s_briza@yahoo.com; 6 Department of Immunology, National Peripheral Histocompatibility Center, Hippokration Hospital, 54642 Thessaloniki, Greece; fylaktoumina@gmail.com; 7 Department of Nephrology, Hippokration General Hospital, Aristotle University of Thessaloniki, 54642 Thessaloniki, Greece; pter43@yahoo.gr (G.L.); zoimhts@gmail.com (Z.M.); aikpapag@otenet.gr (A.P.); 8 Division of Transplantation, Department of Surgery, Hippokration General Hospital, 54642 Thessaloniki, Greece; nikanton@auth.gr

**Keywords:** immunology, inflammation, calcification, atherosclerosis, chronic kidney disease

## Abstract

*Background and Objectives*: recent studies suggest an implication of immune mechanisms in atherosclerotic disease. In this paper, the interaction between inflammation, calcification, and atherosclerosis on the vessel walls of patients with chronic kidney disease (CKD) is described and evaluated. *Materials and Methods*: patients with stage V CKD, either on pre-dialysis (group A) or on hemodialysis (HD) for at least 2 years (group B), in whom a radiocephalic arteriovenous fistula (RCAVF) was created, were included in the study. The control group included healthy volunteers who received radial artery surgery after an accident. The expressions of inflammatory cells, myofibroblasts, and vascular calcification regulators on the vascular wall were estimated, and, moreover, morphometric analysis was performed. *Results*: the expressions of CD68(+) cells, matrix carboxyglutamic acid proteins (MGPs), the receptor activator of nuclear factor-kB (RANK) and RANK ligand (RANKL), and osteoprotegerin (OPG), were significantly increased in CKD patients compared to the controls *p* = 0.02; *p* = 0.006; *p* = 0.01; and *p* = 0.006, respectively. In morphometric analysis, the I/M and L/I ratios had significant differences between CKD patients and the controls 0.3534 ± 0.20 vs. 0.1520 ± 0.865, *p* = 0.003, and 2.1709 ± 1.568 vs. 4.9958 ± 3.2975, *p* = 0.03, respectively. The independent variables correlated with the degree of vascular calcification were the intensity of CD34(+), aSMA(+) cells, and OPG, R^2^ = 0.76, *p* < 0.0001, and, with intima-media thickness (IMT), the severity of RANKL expression R^2^ = 0.3, *p* < 0.0001. *Conclusion*: atherosclerosis and vascular calcification in CKD seem to be strongly regulated by an immunological and inflammatory activation on the vascular wall.

## 1. Introduction

The development of atherosclerosis, arteriosclerosis, and vascular calcification can represent the most important complications of chronic kidney disease and the main reason for the dramatic increase in cardiovascular mortality [1,2,3]. Compared to the general population without CKD, patients with CKD have a 20-fold higher prevalence of early arterial atherosclerosis [4]. Studies show that both accelerated atherosclerosis and increased incidence of cardiovascular disease are associated with a reduction in the glomerular filtration rate [4,5].

The pathogenesis of their development and progression has been the subject of extensive research for more than 150 years. The theory of minor endothelial damage leading to the onset of atherosclerosis was initially developed as early as 1852, while the role of chronic inflammation and LDL oxidation was emphasized some years later [6,7]. After extensive research, the prevailing pathogenic mechanisms of atherosclerosis are anticipated to be instigated by the activation of endothelial cells, including the expression of adhesion molecules and the accumulation of platelets and monocytes, which migrate to the subendothelial region and transform into macrophages [6,8,9].

Early histologic lesions in atherosclerosis are characterized by endothelial cell damage, followed by the migration of vascular smooth muscle cells to the intima, where they proliferate and produce extracellular matrix proteins, leading to diffuse intimal thickening. The transformation of vascular smooth muscle cells into osteoblasts, chondrocytes, and adipocytes, and the infiltration by activated macrophages and lymphocytes, are two parameters which result in the hypertrophy of the media [10,11]. Although atherosclerosis progresses gradually with age and is, thus, often described as a “normal” phenomenon, there are chronic diseases which can accelerate its progression, and chronic kidney disease is the most common condition, followed by accelerated atherosclerosis [4,5,12]. Chronic inflammation and immunological and metabolic disorders in association with physical factors, including hypertension and shear stress, seem to aggravate endothelial damage and force its progression [13].

Meanwhile, however, vascular calcification, another phenomenon directly driven by metabolic factors in CKD, seems to take place simultaneously. Vascular calcification, as a result of mineral deposition on the vascular wall, is not a passive consequence of aging, but, rather, it seems to progress as an active phenomenon determined by metabolic disturbances, hemodynamics, and also by genetic factors [14,15]. Metabolic disorders common in CKD, including hyperparathyroidism or calcium and phosphate abnormalities, act in collaboration with inflammatory and immunological alterations in these patients, resulting in a progressive process of vascular calcification. Previous studies have defined two distinct types of vascular calcification based on their location: in the first type, minerals are deposited in the intima which is associated with the development of atherosclerotic lesions, leading to atherosclerotic calcification, while the second type, more common in CKD patients, is characterized by the calcification of the medial layer [16,17,18].

The pathogenesis of calcification in CKD patients resembles that of atherosclerosis. Both phenomena are consequences of renal dysfunction, the accumulation of uremic toxins, oxidative stress, proinflammatory cytokines, and the activation of several pathways. Vascular smooth muscle cells are stratified, as well as lymphocytes and macrophages, leading to endothelial damage and the progressive development of secondary fibrosis and calcification [9,10,13]. Several factors are responsible for the regulation of the course of vascular calcification, such as the receptor activator of nuclear factor-kB (RANK) and its ligand (RANKL) acting as accelerators, or the matrix carboxyglutamic acid protein (MGP) and osteoprotegerin (OPG) behaving as inhibitors [1,9,10,11,12,13,14,15,16,17,18,19,20,21].

In the present study, we describe the histological changes to the vascular wall of radial arteries in CKD patients, including the assessment of cell proliferation and vascular calcification. Furthermore, we evaluate the local mechanisms which seem to participate in this process, regarding the expression of calcification regulators and the activation of immune mechanisms and pro-inflammatory pathways.

## 2. Materials and Methods

Patients with CKD stage V, undergoing formation of radiocephalic arteriovenous fistula (RC-AVF), were included in the study. Patients were divided into two groups, group A included pre-dialysis CKD-stage V patients, being prepared to start on hemodialysis (HD), and group B, CKD patients who had already been on HD for at least 2 years.

Inclusion criteria were: all patients were Greek-Caucasians, age 25–80 years, gradual deterioration of renal function up to stage V or under dialysis for more than 2 years. All patients should have been under close follow up for at least 3 years prior to enrolment, with adequate control of diabetes, hypertension, dyslipidemia, secondary hyperparathyroidism, and anemia. The vessels used for RC-AVF creation should be intact, meaning that patients should not have a previous attempt for a RC-AVF on the same vessels or the same limp. Exclusion criteria were: active infection, malignancy, autoimmune, or chronic inflammatory disease, previous corticosteroid or immunosuppressive treatment during the last 12 months, and finally, prior attempt or RC- or branchiocefalic (BC)-AVF creation at the same limp.

The control group included healthy volunteers of similar age, sex, and ethnicity, who agreed to have a biopsy from the radial artery during an orthopedic procedure. The control group had no past medical history, or any other surgery in the same limb. All patients were informed and signed the consent form.

The histological characteristics, inflammatory activation and immunophenotypic alterations of the radial artery wall were estimated and their association with the severity of calcification and atherosclerosis were studied.

### 2.1. Histology of the Radial Artery

Radial artery biopsy, obtained during the RC-AVF creation or an orthopedic operation should include a cross-section of the vessel. The biopsy sample was fixed in 10% formaldehyde, dehydrated in alcohol and xylene solutions and encapsulated in a paraffin cube until evaluation. For the optical microscopy evaluation, the paraffin cube was cut into 4 μm sections, deparaffinized, dehydrated, and stained with hematoxylin-eosin (HE) and GT for the assessment of general morphology, evaluation of cell structures and morphometric analysis, and with von Kossa and Verhoff’s elastic methods for the assessment of calcifications. The stained sections were examined under microscope ZEISS Axiolab 5 with the microscope camera Axiocam 208 color.

Vascular calcification: The degree of mineralization was classified semi-quantitatively, in a scale of 0 to 2, where 0 represented no mineral deposition, 1: dispersed concretions, and 2: granular diffuse mineral deposits.

Immunohistochemical analysis: Immunohistochemical analysis was performed to evaluate (i) inflammatory infiltration by monocytes-macrophages, T and B lymphocytes, (ii) phenotypic changes of endothelial cells and smooth muscle cells, and (iii) expression of factors associated with calcification on the vascular wall. The monoclonal antibodies used were:

1. CD3 DAKO monoclonal mouse antibody, clone F7.2.38, code number M7254, dilution 1/100;
2. CD20 THERMO monoclonal mouse antibody L26, dilution 1/100;
3. CD68 THERMO monoclonal mouse antibody KP1, dilution 1/100;
4. CD34 LEICA monoclonal antibody, dilution 1/100;
5. ASMA DAKO mAb clone 1A4 and promoters, dilution 1/200;
6. Receptor activator of Nuclear factor—kB Ligand, (RANKL) TRANCE/TNFSF/RANK L Antibody (12A668), monoclonal antibody, Novus Biologicals, dilution 1/50;
7. Matrix carboxyglutamic acid protein (MGP), Antibody (OTI11G6), monoclonal antibody, Novus Biologicals, dilution 1/50;
8. Osteoprotegerine (OPG)/TNFRSF11B Antibody (98A1071), monoclonal antibody, Novus BiologicalsMatrix carboxyglutamic acid protein dilution 1/100.

Reagents were applied by using a semi-automated system. Primary antibodies were diluted in 1% BSA. Dilutions were arranged and determined after testing for a range of dilutions. Horseradish peroxidase (HRP) conjugated antibodies were applied, slides were incubated in 0.3% H_2_O_2_ in TBS for 15 min, and, finally, the reaction developed in diaminobenzidine (DAB).

For each antibody there was a positive and a negative control.

Morphometric analysis: Morphometric analysis was performed using Image J software (National Institutes of Health) program for windows. On radial artery cross sections, lumen (L), intimal (I), and medial (M) areas were estimated, and, also, the ratios, luminal/intimal (L/I), luminal/medial (L/M), and intimal/medial (I/M) areas were calculated. All measurements were performed on sections stained with GT.

Clinical and Laboratory assessment:

Patients’ history, primary disease and comorbid conditions, medication and clinical examination were recorded based on hospital outpatients’ files. Prior to the scheduled day of RC-AVF creation, all patients underwent laboratory examination, included hematological and serum biochemical analyses.

### 2.2. Carotid Artery Intima—Media Thickness (IMT) Assessment

Presence and severity of atherosclerotic lesions in CKD patients was assessed based on the measurement of common carotid intima—media thickness (IMT) of the common and internal carotid on both sides. The measurements were made using an Aloka Sonos SSDE—1700 ultrasound scanner and a 7.5 MHz high-resolution head and were performed by the same radiologist who was aware of patients’ clinical and laboratory data. The thickness of the intermeddle tunica corresponded to an ultrasound gray zone which does not project into the arterial lumen.

The determination of IMT was performed at six points, 0.5, 1, and 2 cm above the bifurcation of the common carotid artery (in the internal carotid artery) and 0.1, 1, and 2 cm below the bifurcation of the common carotid artery, on both sides, in areas without lesions, and the average value of the above measurements was set as IMT. In patients undergoing dialysis, measurements were performed on a day between dialysis sessions.

### 2.3. Life Expectancy of RC-AVF

The patients were followed for one year with clinical and laboratory testing every three months. During that period the clinical and laboratory indices of atherosclerosis and cardiovascular diseases were evaluated and the maturation and complications of arteriovenous fistulas were recorded.

### 2.4. Statistics

Package for Social Sciences (SPSS Inc., Chicago, IL, USA) for windows, version 25.0 was used for the Statistical analysis. *p* values < 0.05 (two-tailed) were considered statistically significant for all comparisons. Shapiro–Wilk and/or Kolmogorov–Smirnov tests were applied to determine normality of variables. Normal distributed continuous variables were expressed as mean ± standard deviation, while data from non–parametric variables were expressed as medians and range. Differences between groups were estimated by Student’s t-test for independent samples, for normally distributed variables, and Mann–Whitney U test, Wilcoxon signed ranks test, and Kruskal–Wallis H test were used for non-parametric variables.

Multiple regression analysis was performed to estimate independent variables correlated by a dependent parameter.

## 3. Results

### 3.1. Patients’ Characteristics

Fifty patients with chronic kidney disease (CKD), stage V, either pre-dialysis (*p* = 25) (group A) or on hemodialysis (HD) (*p* = 25) (group B) were included in the study. Clinical and laboratory characteristics of patients at time of AVF formation are depicted on Table 1 and Table 2, respectively. There were no significant differences in age, sex, race, and also in the frequency of dyslipidemia, diabetes mellitus, or smoking habits between patients and controls.

IMT was significantly increased in patients, compared to controls, 0.6 (0.22–1.2) mm vs. 0.26 (0.18–0.51) mm, respectively, *p* = 0.003.

### 3.2. Inflammatory Infiltration and Activation of Vascular Cells

Vascular infiltration by CD3(+) and CD20(+) cells was slightly increased in the whole cohort of CKD patients compared to controls, mean rank of 22.49 vs. 19 and 23 vs. 15.837, respectively, but these differences did not reach statistical significance. In a similar way, the severity of a-SMA expression was increased in CKD patients, but not statistically significant, mean rank of 22.97 vs.16, *p* = NS. The intensity of CD68(+) cell infiltration was significantly increased in CKD patients compared to healthy controls, mean rank of 23.3 vs. 13.42, *p* = 0.02.

Comparing the expression of the above parameters between group A, group B, and healthy controls, there was a propensity for a gradually increased concentration of inflammatory cells, though not reaching statistical significance. Table 3 summarizes differences in the severity of infiltrating cells between three groups.

### 3.3. Vascular Calcification (VC)

#### 3.3.1. Differences in the Severity of VC between CKD Patients and Controls

**Figure 1:**
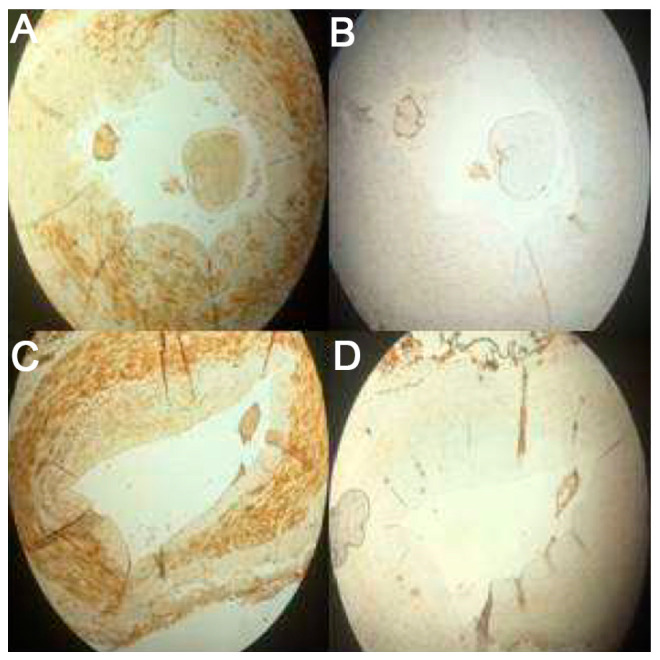
Immunohistochemistry on the radial artery wall, showing expression of OPG (**A**,**C**) and RANKL (**B**,**D**) of healthy controls and CKD patients, respectively.

Severity of VC was evident in hematoxylin staining, however, specific evaluation was performed by applying von Kossa and Verhoff’s Elastic staining (Figure 1). Thirty two patients (64%) had positive von Kossa staining, compared to 13 (65%) of controls, *p* = NS, however, the severity of calcification had significant differences between patients and controls,16 (32%), 23 (46%), and 11 (22%) of CKD patients were classified as grade 0, 1, and 2, respectively, and 7 (35%), 13 (65%), and 0 of controls as grade 0, 1, and 2, respectively, Chi-square 7.9, *p* = 0.01.

Similarly, based on the Verhoff’s Elastic staining, 13 (26%), 32 (64%), 5 (10%) of CKD patients had grade 0, 1, and 2 calcification, respectively, compared to 10 (50%), 10 (50%), and 0 of controls, Chi-square 5.9, *p* = 0.01.

The severity of RANKL, MGP, and OPG expression was estimated in the two groups of patients, and results were compared to controls. There were significant differences between the whole cohort of patients and controls, regarding the expression of RANKL (Mean rank 21.56 vs.8.5, *p* = 0.006), MGP (Mean rank 21.47 vs. 9, *p* = 0.01), and OPG (Mean rank 21.56 vs. 8.5, *p* = 0.006). Differences in the expression of these parameters between the three groups were also significant and are shown at Table 3 and Figure 1.

#### 3.3.2. Association between Calcification Regulators, Inflammation and Cellular Activation with VC Intensity

Expression of calcification regulators, namely RANKL, MGP, and OPG was correlated significantly with the degree of infiltration by CD3(+), CD20(+), and CD68(+) cells, as well as the expression of CD34(+) and α-SMA(+) cells, as depicted at Table 4. Significant correlation was found between inflammatory infiltration (expression of CD3(+), CD20(+), CD68(+) cells), cellular activation (CD34(+), a-SMA(+) cells) and calcification regulators (MGP, RANKL, OPG) with the degree of vascular calcification, as this was estimated and classified based on Verhoff’s elastic and von Kossa staining (Table 5).

In multiple regression analysis, independent variables for the severity of vascular calcification were the intensity of CD34(+) cells (B = 0.595, *r* < 0.0001), aSMA(+) cells (B = 0.454, *p* = 0.004), and OPG (B = −223, *p* = 0.01), R^2^ = 0.76, *p* < 0.0001.

### 3.4. Atherosclerotic Lesions

#### 3.4.1. Morphometric Changes of Radial Artery

**Figure 2:**
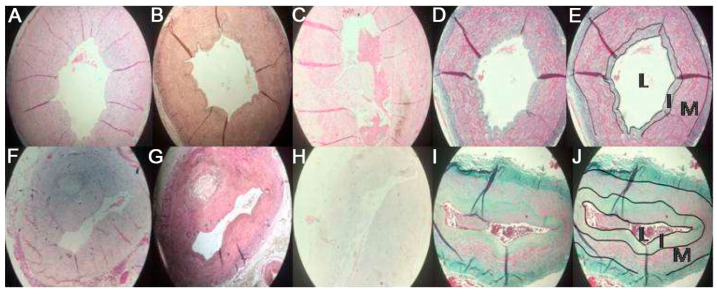
Histology and morphometric analysis of the radial artery samples. The upper panel represents the control group, and the lower panel represents patients. Hematoxylin (**A**,**F**), Verhoff’s elastic (**B**,**G**), Von Kossa (**C**,**H**) and GT staining (**D**,**I**) of the radial artery wall controls and patients, respectively. Morphometric analysis on GT staining (**E**,**J**) of controls and patients, respectively, showing luminal (**L**), intimal (**I**), and medial (**M**) areas.

Significant differences between patients and controls were noticed in the ratios intimal/media (I/M) and luminal/intimal (L/I), which strongly indicated that intimal enhancement predominated in CKD patients, resulting in luminal stenosis (Figure 2). The I/M ratio was significantly increased in CKD patients compared to controls, 0.3534 ± 0.20 vs. 0.1520 ± 0.865, *p* = 0.003. Accordingly, L/I ratio in CKD patients and controls was 2.1709 ± 1.568 vs. 4.9958 ± 3.2975, respectively, *p* = 0.03, while luminal/medial (L/M) ratio was similar, 0.5310 ± 0.2417 vs. 0.7830 ± 0.2044, respectively, *p* = NS.

No significant differences were evident in either I/M, L/I and L/M between group A and group B patients.

#### 3.4.2. Correlation of IMT with the Severity of Inflammation, Endothelial Activation, and Vascular Calcification

CKD patients had significantly elevated IMT compared to controls, 0.6 (0.22–1.2) vs. 0.26 (0.18–0.51), mean rank 21.7 vs. 7.75, respectively, *p* = 0.003. Significant changes in IMT were evident between controls 0.26 (0.18–0.51), Group A (pre-HD) patients 0.6 (0.22–1.2) and Group B (HD) patients 0.62 (0.32–1.1) (Table 2).

**Figure 3:**
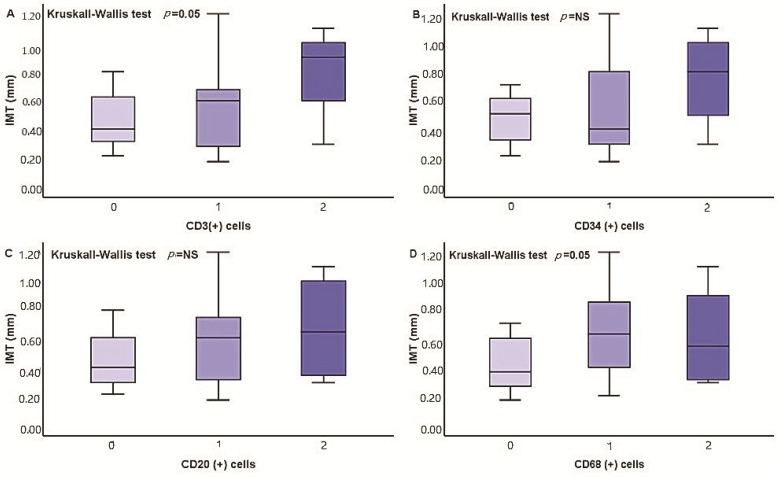
Differences in IMT cells according to the severity of radial artery infiltration by CD3(+) (**A**), CD34(+) (**B**), CD20(+) (**C**), and CD68(+) (**D**) cells.

**Figure 4:**
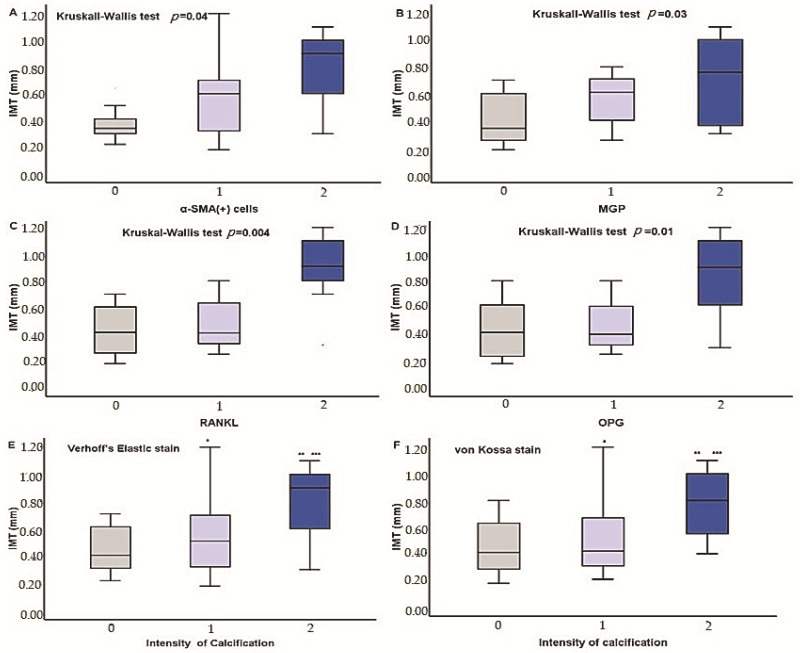
Differences in IMT cells according to the severity of radial artery infiltration by a-SMA(+) cells (**A**), MPG (**B**), RANK (**C**), OPG (**D**), and intensity of calcification based on Verhoff’s elastic (**E**) and von Kossa (**F**) stain.

IMT had significant positive correlation with the intensity of aSMA(+) cells, *r* = 0.3, *p* = 0.03, CD68(+) cells, *r* = 0.3, *p* = 0.03, expression of MGP, *r* = 0.4, *p* = 0.007, RANKL, *r* = 0.4, *p* = 0.002, OPG, *r* = 0.5, *p* = 0.004, and, also, with iPTH levels, *r* = 0.4, *p* = 0.02 and triglyceride serum levels, *r* = 0.5, *p* = 0.002. The association of IMT levels with the severity of inflammatory infiltration, expression of calcification regulators, and calcification severity is shown on Figure 3 and Figure 4.

In multiple regression analysis however, the only independent factor correlated with IMT levels was the severity of RANKL expression, B = 0.198, R^2^ = 0.3, *p* < 0.0001.

### 3.5. AVF Survival

At the end of the one year follow up, 14 (28%) of AVFs performed had failed. Patients with failed AVF had no significant differences in the frequency of comorbid conditions, such as hypertension, diabetes mellitus, cardiovascular disease, and also no differences in the severity of inflammatory infiltration, calcification, and calcification regulators. The only parameter which was significantly increased in patients in whom AVF failed was the I/M ratio, 0.38 ± 0.22 vs. 0.26 ± 0.12, *p* = 0.04, suggesting that the intimal thickening was the main factor regulating survival of AVF and implicated in its failure.

## 4. Discussion

Vascular calcification, as well as development of atherosclerosis are common consequences in chronic kidney disease [1,18]. Although metabolic and hemodynamic disorders are considered to predominate in the pathogenesis of both conditions, recent accumulating evidence emphasizes the causative role of immune mechanisms. Immunological disorders in CKD are usually the result of impaired renal function per se, which seems to affect both cell and humoral immunity, leading to alterations in T and B cell phenotype and activation of inflammatory cells, phenomena implicated in the development of cardiovascular disease [22,23].

In the present study, we evaluated the severity of vascular calcification, as it is evident on the wall of medium sized arteries in CKD patients, either at pre-dialysis stage or after being on hemodialysis for more than three years. We also performed morphometric analysis on the biopsies of radial arterial walls to estimate the degree of lumen stenosis and, also, expansion of intimal and medial areas. We evaluated the possible implication of local inflammatory and immunological mechanisms, activation in atherosclerotic changes and calcification of the vascular walls, and, finally, we assessed correlation between atherosclerotic and calcification pathways.

Severity of vascular calcification, as this was estimated by both von Kossa and VE methods, was significantly increased in CKD patients compared to controls, with no difference between pre and post-dialysis patients. Similarly, expression of the calcification regulators, RANKL, MGP, and OPG was significantly increased in CKD patients and the severity of their staining showed positive correlation with the degree of vascular calcification, and, also, with the intensity of macrophage, myofibroblast, T and B cell infiltration, and endothelial cell activation. Interestingly however, although RANKL, MGP, and OPG were significantly increased in CKD, there were no significant differences in the expression of inflammatory and activation indices; suggesting either that even a trivial activation of macrophages and lymphocytes can stimulate production of calcification regulators, or that other factors influence this process.

The role of macrophages in the progress of vascular calcification is rather complicated, as they can either promote its process, through the release of reactive oxygen species, matrix vesicles, and pro-inflammatory cytokines, or suppress it, through production of anti-inflammatory factors and differentiation into osteoclast-like cells [24]. Migration of monocytes to the sub-endothelial space seems to be the critical step, followed by their differentiation into dendritic cells and macrophages, which interact with vascular wall cells, through releasing pro-inflammatory factors (TNF, IL-1, IL-6). Osteogenic genes, such as runt-related transcription factor 2 (Runx2), OPN, and bone morphogenetic protein 2 (BMP-2), are also expressed and released by macrophages and stimulate the osteogenic process [24,25,26]. The significant correlation between RANKL, OPG, and MGP expression with macrophage infiltration, described in our study, support this role of macrophages, but, furthermore, positive correlation with α-SMA expression indicates a synergistic activity of RANK/RANKL/OPG pathway with myofibroblast formation and smooth muscle cell calcification. 

The description of RANKL/RANK/OPG pathway particularly elucidates the progress of bone metabolism, as well as vascular wall changes during calcification [27,28]. RANKL is a transmembrane protein expressed on T lymphocytes, osteoblasts, endothelial cells, and, also, on vascular smooth muscle cells at calcification sites [19]. Its soluble form binds to its receptor, RANK, a transmembrane protein expressed on dendritic cells and osteoclasts [20,29]. Only calcified arteries express RANKL and RANK, stained on areas of calcification, while normal tissues express OPG [30,31] and MGP, a protein protective against calcification, mainly expressed by vascular smooth muscle cells [32,33]. OPG is secreted protein, an “atypical” member of the TNF family, with no transmembrane region and no direct signal transduction properties. OPG acts as a soluble RANKL receptor, inhibiting the RANK–RANKL reaction and thereby inhibiting the calcification process [34,35]. Interestingly, recent experimental and clinical studies highlighted the multifactorial effect of OPG, as elevated serum levels appear to inhibit calcification, while at the same time being associated with hyperlipidemia, atherosclerosis, and increased cardiovascular risk [35,36].

In our study, the degree of OPG staining, together with the degree of CD34 and a-SMA expression, were the only independent parameters correlating to the degree of vascular calcification, indicating that endothelial cell activation, myofibroblast formation and OPG pathway are mainly implicated in the development of vascular calcification in CKD patients.

We also described a close correlation between classification and atherosclerotic changes in our patients. In most studies, early atherosclerotic lesions are lost, as assessment of atherosclerotic changes is almost exclusively based on imaging methods, which illustrate merely advanced vascular lesions. In the present study, we performed histo-morphometric analysis to assess early atherosclerotic changes, and we also estimated the IMT in order to evaluate the severity of established atherosclerotic lesions. Both morphometric analysis and IMT calculation showed a significant expansion of intimal area, which, according to increased I/M and reduced L/I ratio, described in morphometric measurements, seems to lead to lumen stenosis, and finally, to the failure of AVF.

Our findings confirm previous studies which showed a neointimal hyperplasia, as the dominant histologic finding in CKD patients [37]. We did not find any correlation between I/M and IMT. It could be attributed to different pathogenetic pathways activated in middle size arteries, such as radial artery, where morphometric analysis was performed, and in large size arteries, such as carotid artery. However, we rather believe that additional parameters may be implicated during progress of CKD.

Levels of IMT correlated with most inflammatory indices and calcification regulators, most important factors being the severity of RANKL staining, a-SMA, and CD68 infiltration, however, in multiple regression analysis, intensity of RANKL was the only independent parameter correlated with IMT, and this finding further supports the correspondence between calcification and atherosclerotic pathways in our patients.

The role of the effect of lymphocytes on the appearance and development of atherosclerosis, although initially disputed, is beginning to gain particular interest in recent years [22,23]. Recent studies indicated immune mechanisms, such as activation of complement or specific T and B lymphocyte subpopulations to play a significant role in the atherosclerotic process [38]. Different subpopulations of T lymphocytes have different effects on atherosclerosis, Th1, Th17 cells, natural killer cells (NKT), CD28 null cells promote the process of atherosclerosis, while some of them are expressed on atherosclerotic plaques [22]. In contrast, Th2, FoxP3^+^nTregs, Bregs exert a protective effect, possibly through the secretion of IL-10, TGF-β and antibodies against oxidized LDL (anti-oxLDL antibodies) [39,40].

## 5. Conclusions

In the present study, we performed histo-morphometric analysis to describe and evaluate early vascular lesions in CKD patients and we subsequently, compared results with established imaging methods assessing advanced atherosclerosis. We proved a close relationship between histopathological pathways of atherosclerosis and vascular calcification in CKD patients. Vascular infiltration of lymphocytes and macrophages, activation of endothelial cells, myofibroblast formation and activation of RANKL/RANK/OPG pathway are important to both phenomena, which seem to have a parallel and interactive progression.

## Figures and Tables

**Table 1:** Clinical characteristics of patients and controls. Differences between group A (pre-HD), group B (HD) patients.

	Controls	All Patients	Group A	Group B	*p* (*Group A vs. B*)
*n*	20	50	25	25	
Age (yrs)	60.3 ± 12.3	64 ± 12.4	63.7 ± 12.6	64.8 ± 12.5	NS
M/F	12/8	27/23	14/11	13/12	NS
**Primary Disease**					
Diabetes Mellitus (%)	3 (15)	17 (34)	9 (36)	8 (32)	NS
Systemic Disease	0	8 (16)	5 (20)	3 (12)	0.03
Glomerulonephritis (%)	0	12 (24)	7 (28)	5 (20)	NS
Cystic disease (%)	0	5 (10)	2 (8)	3 (12)	NS
Other (%)	0	8 (16)	3 (12)	5 (20)	NS
**Comorbidities**					
Coronary Artery Disease (%)	0	5 (10)	2 (8)	3 (12)	NS
Hypertension (%)	5 (25)	38 (76) *	18 (72)	20 (80)	NS
Dyslipidemia (%)	4 (20)	10 (20)	6 (24)	4 (16)	NS
Secondary Hyperparathyroidism (%)	0	31 (62)	15 (60)	16 (64)	NS
Smoke (%)	5 (25)	18 (36)	11 (44)	7 (28)	NS
**Medication**					
Ca-channel blockers (%)	4 (20)	22 (44)	18 (72)	4 (16)	<0.0001
RAAS inhibitors (%)	3 (15)	6 (12)	2 (8)	4 (16)	NS
Diuretics (%)	3 (15)	25 (50) **	22 (88)	3 (12)	<0.0001
B-blockers (%)	1 (5)	10 (20)	7 (28)	3 (12)	NS

* *p* (patients vs. controls) < 0.0001, ** *p* (patients vs. controls) = 0.006.

**Table 2:** Laboratory findings from group A (pre-HD), group B (HD) patients and controls at time of investigation.

	Controls	All Patients	*p*	Group A	Group B	*p*
*n*	20	50	Patients vs. Controls	25	25	Group A vs. B
SerumUrea (mg/dL)	42.7 ± 2.4	91.4 ± 45.8	0.005	88.8 ± 12	142.3 ± 13.2	0.01
Serum Creat (mg/dL)	0.9 ± 0.1	3.8 ± 3.2	<0.0001	3.7 ± 2.5	6.8 ± 2.7	0.003
Serum Glucose (mg/dL)	93 ± 21	97.6 ± 22	NS	81 ± 15.5	103.9 ± 14.8	NS
Serum Ca (mg/dL)	9.8 ± 0.2	9.5 ± 0.5	0.04	9.6 ± 0.5	9.23 ± 0.3	0.04
Serum *p* (mg/dL)	4.70.5	4.9 ± 0.7	NS	4.8 ± 0.7	4.92 ± 0.7	NS
Cholesterol (mg/dL)	191.2 ± 27.8	206.9 ± 29.9	NS	208 ± 29	203 ± 32	NS
Triglycerides (mg/dL)	46.8 ± 9.2	150.0 ± 64.7	<0.0001	152.8 ± 66	139.9 ± 61	NS
HDL (mg/dL)	46.3 ± 6.8	56.1 ± 17.2	NS	54.3 ± 16	62.6 ± 1+	NS
LDL (mg/dL)	99.3 ± 20.1	101.0 ± 18.0	NS	97.5 ± 18	112.1 ± 12	0.02
Serum Albumin (g/dL)	3.9 ± 0.3	3.8 ± 0.5	NS	3.8 ± 0.4	3.9 ± 0.5	NS
CRP (mg/dL)	1.9 ± 0.7	1.24	NS	1.2 ± 0.6	1.3 ± 0.8	NS
intact PTH (pg/mL)	29 ± 12	190.1	<0.0001	198 ± 76	288 ± 130	0.02
IMT	0.26 ± 0.1	0.6 ± 0.3	0.003	0.56 ± 0.2	0.63 ± 0.3	NS

**Table 3:** Severity of radial artery calcification based on Verhoff’s elastic and von Kossa stain, cellular infiltration and expression calcification regulators on the radial artery. Differences between group patients and controls are expressed by mean ranks and estimated by Kruskal–Wallis test.

	Controls	Group A	Group B	*p* (Kruskal-Wallis Test)
**Calcification (Verhoff’s elastic stain)**	15.25	20.32	20.21	NS
**Calcification (von Kossa stain)**	19.33	19.12	21	NS
**CD3(+) cells**	17.25	19.44	21.64	NS
**CD20(+) cells**	15.17	19.82	22.07	NS
**CD34(+) cells**	19.83	18.9	21.36	NS
**CD68(+) cells**	13.17	19.62	24.5	NS
**α-SMA(+) cells**	13.75	20.28	21.64	NS
**MGP**	9	20.7	24.21	0.01
**RANKL**	8.5	20.18	26.5	0.006
**OPG**	8.5	19.98	27.21	0.005

**Table 4:** Correlation between inflammatory infiltration and vascular calcification regulators.

	RANKL		MGP		OPG	
	*r*	*p*	*r*	*p*	*r*	*p*
CD3(+) cells	0.36	0.02	0.42	0.008	0.33	0.04
CD20(+) cells	0.45	0.004	0.59	<0.0001	0.54	<0.0001
CD68(+) cells	0.73	<0.0001	0.81	<0.0001	0.74	<0.0001
CD34(+) cells	0.51	0.001	0.5	0.001	0.51	0.001
α-SMA(+) cells	0.4	0.01	0.59	<0.0001	0.46	0.004

**Table 5:** Differences on the expression of CD3(+), CD20(+), CD68(+), a-SMA(+) cells, and MGP, RANKL, and OPG according to the severity of radial artery calcification.

	CD3	CD20	CD68	CD34	α-SMA	MGP	RANKL	OPG
**Verhoff’s Elastic stain**								
0	12.88	10.92	13.17	11.33	12.29	14.88	14.96	16.67
1	20.67	21.76	20.72	21.54	20.98	19.96	20.33	19.09
2	37	36.5	35.5	36.5	37	34.5	31.33	34
*p*	0.001	<0.0001	0.002	<0.0001	<0.0001	0.012	0.04	0.034
**Von Kossa stain**								
0	16.67	17.43	14.77	15.3	17.83	17.7	16.77	16.97
1	19.21	18.08	19.87	19.8	18.66	18.32	18.97	19.03
2	31.5	34	35.5	33.75	29.75	31.88	32.25	31.25
*p*	0.029	0.009	0.001	0.005	0.056	0.039	0.027	0.048

## Data Availability

The data presented in this study are available on request from the corresponding author. The data are not publicly available due to privacy.
